# Adverse drug reactions reported by consumers for nervous system medications in Europe 2007 to 2011

**DOI:** 10.1186/2050-6511-14-30

**Published:** 2013-06-13

**Authors:** Lise Aagaard, Ebba Holme Hansen

**Affiliations:** 1 Clinical Pharmacology, Institute of Public Health, Faculty of Health Sciences, University of Southern Denmark, J.B. Winsløws Vej 19, Odense C, DK - 5000, Denmark; 2 Section for Social and Clinical Pharmacy, Department of Pharmacy, Faculty of Health and Medical Sciences, University of Copenhagen, Copenhagen, Denmark; 3 Danish Pharmacovigilance Research Project (DANPREP), Copenhagen, Denmark

**Keywords:** Adverse drug reactions, Nervous system medications, Pharmacovigilance, Consumers, EudraVigilance

## Abstract

**Background:**

Reporting of adverse drug reactions (ADRs) has traditionally been the sole province of healthcare professionals. In the European Union, more countries have allowed consumers to report ADRs directly to the regulatory agencies. The aim of this study was to characterize ADRs reported by European consumer for nervous system medications.

**Methods:**

ADRs reported by consumers for nervous system medications (ATC group N) from 2007 to 2011 and located in the European ADR database, EudraVigilance, were analysed. Data were categorized with respect to age and sex, category and seriousness of reported ADRs and medications. The unit of analysis was one ADR.

**Results:**

We located 4766 ADRs reported for nervous system medications, and one half of these were serious including 19 deaths. Less than 5% of ADRs were reported in children. Totally, 58% of ADRs were reported for women, 42% for men. The majority of reported ADRs were of the types “nervous system disorders” (18% of total ADRs) followed by “psychiatric disorders” (18% of total ADRs) and “general disorders” (15% of total ADRs) which also were the system organ classes in which the majority of serious ADRs were found. ADR reports encompassed medicines from the therapeutic groups: antiepileptics (ATC group N03) (36% of total ADRs), parasympathomimetics (ATC group N07) (22% of total ADRs) and antidepressants ATC group N06A (9% of total ADRs). Antiepileptics were the therapeutic group with the highest share of serious ADRs (60%) followed by antidepressants (15%). Many serious ADRs were reported for pregabalin and varenicline.

**Conclusions:**

The majority of ADRs from nervous system mediations reported by consumers that were identified from the EudraVigilance database were serious. The value of consumer reports in pharmacovigilance still remains unclarified.

## Background

Reporting of adverse drug reactions (ADRs) to national databases has traditionally been the sole province of health care professionals [1]. In order to strengthen the systems in some countries, consumers have also been allowed to report ADRs directly to the regulatory agencies [2]. Consumers can provide first-hand information about their experience with medicines and may therefore constitute a valuable information source [1,2]. The weakness of consumer ADR reports is the lack of medical confirmation, which might impede the interpretation of ADR causation [2]. Only few studies have analysed consumer reports submitted to ADR databases, but over the last years studies analysing ADRs reported to national pharmacovigilance databases have been published [3,4]. Medawar and Herxheimer investigated ADR reports on the risk of dependence and suicidal behaviour from paroxetine from UK consumers and healthcare professionals, respectively [5]. In 2011, McLernon et al. published a study investigating the characteristics of consumer ADRs reported in UK from 2008 to 2009 [6]. In Sweden, it has been possible for consumers to ADR report directly to the non-profit organization KILEN since 1978 [2], and research conducted on these data has been published in several papers and reports [7-10]. Experience with consumer reporting (2004 to 2007) in the Netherlands was recently published showing differences in the categories of seriousness and outcome of the reported ADRs between patients and healthcare professionals [11]. A study from Denmark analysing differences in ADR reporting patterns between consumers and healthcare professionals (2004 to 2006) showed that patients were more likely to report ADRs from nervous and psychiatric medications, that patients’ share of reports on serious ADRs was comparable to that of physicians, and that patients provided new and unknown information about ADRs [12]. Analysis of consumer reports of suspected ADRs submitted voluntarily to the website of a Danish consumer magazine showed that consumers reported ADRs for nervous systems medications and that patients report rather unspecific symptoms, as they use lay terms to describe reactions [13]. Patients also reported several ADRs, which prescribers may not consider serious but may be troublesome to patients and therefore patients find worthy of reporting [13]. The published consumer studies which all were conducted on national datasets showed that consumers are willing to report many ADRs for nervous system medications, but we do not know to which extent the above findings are generaliserable to populations in other countries. Since 2012, researchers were allowed access to ADR data in the EU ADR database, EudraVigilance (EV) and this has opened for cross-national analysis based on a standardised reporting format [14]. The objective of this study was to investigate ADR reports submitted by consumers for nervous system medications in Europe during the first 5 years of electronic reporting to the EV ADR database.

## Methods

### Setting

EudraVigilance (EV) is the central database of reports of suspected spontaneous ADR reports and ADRs reported in clinical trials for all medicinal products authorized in the European Economic Area (EEA) [15]. In compliance with the EU pharmacovigilance legislation, ADRs are reported to EV by regulatory agencies in member states where the ADR occurred. EV was set up in December 2001 to facilitate the electronic reporting of ADRs in the EEA. Data should be transmitted in accordance with the ICH E2B (R2) standard [15]. The minimum information required for an ADR report to enter the EV database is the following parameters: type of reporter, patient, at least one suspected active substance/medicinal product, and at least one suspected ADR (Volume 9A) [15]. The EV database is not publically accessible, and authorisation for data access was given by the European Medicines Agency. By 2012, consumer reporting was officially accepted in 5 European countries: Denmark, the Netherlands, Norway, Sweden and the United Kingdom [1]. Before July 2012, countries were only requested to forward serious consumer ADR reports to the EV database [16].

### Study design

The study comprised all ADR reports occurring from 2007 to 2011, located in the EV database and reported by consumers for nervous system medications (ATC group N). The content of the reports was analysed with respect to seriousness, categories of ADRs classified by system organ class (SOC) and medications. The unit of analysis was one ADR. Patients’ age was dichotomized into two groups: children (0-17-year-olds) and adults (18 +).

## Material

ADR information was provided for this study in anonymous form with encrypted identification [8]. Data extraction and data analyses of the raw material were comprehensive and time-consuming. Information was extracted from the ADR database on the date reports were received; category of persons submitting the reports; and criteria of seriousness and medications for which the ADRs were reported. The reported ADRs were coded according to type and seriousness using CIOMS (Council for International Organizations of Medical Sciences) criteria by academic staff in the national regulatory agencies [16]. ADR data was placed at the disposal of this study in anonymous form with encrypted identification of the medicine user. Data were extracted from the EV database in Microsoft Excel files using the following criteria: patient’s sex and age, medicines (active substance), adverse drug reaction and severity. EMA has to ensure that, in complying with regulation (EC) 1049/2001, the protection of privacy and integrity of individuals is guaranteed, and therefore individual country specific ADR information was not disclosed [17]. The material comprised all ADRs reports from consumers reported to the EV database from 2007 to 2011. Data were extracted from the EV database and delivered to us as several large Excel files. Data comprised all ADR reports form consumers located in the EV database by 14 March 2012. In STATA® (statistical software package) the Excel files were merged into one major file and the ADR reports were searched for duplicates. Data analysis including coding of ADR reports was conducted in an Access database. Each ADR report may refer to one or more suspected ADR (s) as well as to one or more medicinal products. In this study we included ADRs reported for medications, which were listed as suspect drug by the reporter, meaning that the reporter suspected this drug and not the concomitant medicine to have caused the ADR.

### Classification of ADRs by type

The different types of reported ADRs were classified according to the *Medical Dictionary for Regulatory Activities* (MedDRA) System Organ Class (SOC) [18]. Serious ADRs were defined as: fatal, life-threatening, requiring hospitalisation or prolongation of existing hospitalisation, resulting in persistent or significant disability/incapacity in the reporter’s assessment, in a congenital anomaly/birth defect and other medically important conditions. All other ADRs are classified as non-serious [18].

### Classification of medications by anatomical therapeutic chemical (ATC) group

The ATC system is a system for classifying medicinal products according to their primary constituent, the organ or system on which they act and their chemical, pharmacological and therapeutic properties [19]. Medicinal products are classified at five different levels. The medicines are divided into 14 main groups (first level), with one pharmacological/therapeutic subgroup (second level), and the fifth level is the chemical substance [18]. As the ADR data provided by EMA did not contain any information about ATC codes, these were added manually to the data file. The medicinal products reported are referenced based on their active substance and in this article we present ADR data at ATC level 1 and 5 [19].

## Results

From 2007 to 2011, a total of 7434 consumer ADR reports containing information about 35349 ADRs was located in EV. Of these, 4766 ADRs were submitted for nervous system medications. In total, 51% of ADRs were classified as serious and of these 19 fatal cases were reported. The characteristics of the fatal cases are displayed in Table 1. The largest number of fatal cases (n = 8) was reported for apomorphine (ATC group N04) followed by five fatal cases reported for antidepressants (ATC group N06). Totally, 58% of ADRs were reported for women and 42% for men. Less than 5% of ADRs were reported in children.

**Table 1:** Fatal consumer cases reported for nervous systems medications in Europe, 2007 to 2011

**Case no.**	**Medicine (s)**	**ATC group**	**Adverse drug reaction (s)**	**Sex (M/F)**	**Age**
1	Diamorphine	N02AA09	Sudden death	F	18+
2	Metamizole	N02BB02	Agranulocytosis	F	18+
			Leukopenia		
			Multi-organ failure		
			Sepsis/septic shock		
3	Morphine	N02AA01	Cerebrovascular accident	F	18+
4	Oxycodone	N02AA05	Intentional overdose/suicidal ideation	M	18+
5	Apomorphine	N04BC07	Pneumonia	M	NA
6	Apomorphine	N04BC07	Intestinal haemorrhage	M	NA
			Pneumonia aspiration		
7	Apomorphine	N04BC07	Anaemia	F	18+
			Haematocrit decreased		
			Red blood cell sedimentation rate increased		
8	Apomorphine	N04BC07	Death	F	NA
9	Apomorphine	N04BC07	Death	F	18+
10	Apomorphine	N04BC07	Death	F	NA
11	Apomorphine	N04BC07	Death	F	18+
12	Carbidopa/levodopa	N04BA02	Death	M	18+
	Entacapone, Rotigotine				
13	Clomethiazole	N05CM02	Leucocytosis	F	NA
			Pyrexia		
			Musculoskeletal stiffness		
			Neuroleptic malignant syndrome		
14	Clozapine	N05AH02	Cardiac failure	F	18+
			Somnolence		
15	Citalopram	N06AB04	Fatigue/malaise	F	NA
16	Duloxetine	N06AX21	Deafness	F	18+
			Abasia		
			Urinary tract infection		
			Septic shock		
			Urosepsis		
			Hyponatraemia		
			Neoplasm malignant		
			Aphasia		
			Urinary incontinence		
			Renal failure		
17	Trimipramine	N06AA06	Asthenia	M	18+
			Depressed level of consciousness/sedation		
			Tachyphrenia		
			Completed suicide		
			Dependence		
			Indifference		
18	Amitriptyline	N06AA09	Toxicity to various agents	M	NA
19	Rivastigmine	N06DA03	Lung infection	F	18+
			Mood altered/aggression		

*M: male, F: female, NA*:no information available.

### ADRs by type and seriousness

Table 2 shows the distribution of reported ADRs by SOC. In total, consumers reported 26 ADR categories. The largest shares of ADRs were reported for the SOCs: nervous system disorders (18% of total ADRs), psychiatric disorders (18% of total ADRs); and general disorders and administration site conditions (15% of total ADRs). The largest share of serious ADRs was of the type psychiatric disorders (23% of serious) followed by nervous system disorders (17% of serious) and ADRs of the general type (12% of ADRs).

**Table 2:** Number of consumer adverse drug reactions for nervous system medications in Europe by type and seriousness, 2007 to 2011

**System organ class (descending order)**	**Number (serious)**
Psychiatric disorders	868(547)
Nervous system disorders	847(424)
General disorders and administration site conditions	736(303)
Gastrointestinal disorders	651(199)
Investigations	251(151)
Skin and subcutaneous tissue disorders	236(118)
Musculoskeletal and connective tissue disorders	219(96)
Injury, poisoning and procedural complications	145(93)
Eye disorders	142(65)
Respiratory, thoracic and mediastinal disorders	123(69)
Cardiac disorders	79(57)
Vascular disorders	74(58)
Metabolism and nutrition disorders	69(44)
Renal and urinary disorders	60(38)
Ear and labyrinth disorder	56(29)
Infections and infestations	50(29)
Reproductive system and breast disorders	44(15)
Blood and lymphatic system disorders	21(21)
Social circumstances	19(14)
Surgical and medical procedures	18(17)
Hepatobiliary disorders	17(15)
Immune system disorders	16(10)
Neoplasm benign, malignant and unspecified	10(10)
Endocrine disorders	9(9)
Congenital, familial and genetic disorders	2(2)
Pregnancy, puerperium and perinatal conditions	2(2)
Total	4766 (2433)

### ADRs by therapeutic groups

Table 3 displays the number of ADRs reported by consumers distributed on therapeutic groups and seriousness. Reports encompassed medicines from the therapeutic groups: antiepileptics (ATC group N03) (36%), parasympathomimetics (ATC group N07) (22%) and antidepressants ATC group N06A (9%). Except from parasympathomimetics, the majority of ADRs were serious. In particular, a large number of ADRs were reported for prebagalin (n = 1510) and varenicline (n = 1017). The most commonly reported ADRs for venlafaxine were anxiety, restlessness, paraesthesia and sleep disorder. Table 4 displays characteristics of serious ADRs reported for pregabaline. In total, 50 ADR categories were reported; the most frequently reported ADRs were drug ineffective/drug effect decreased (n = 83), dizziness (n = 78), pain (n = 61), somnolence (n = 56) and fatigue (n = 54). Table 5 displays the characteristics of serious ADRs reported for varenicline. The largest number of reported ADRs was musculoskeletal pain (n = 9), sleep disorder (n = 7), chest disorder/pain (n = 7), depression (n = 6) and suicidal behavior/ideation (n = 6).

**Table 3:** Consumer adverse drug reactions (N) for nervous system medications in Europe by therapeutic group and seriousness (in parentheses), 2007 to 2011

**Therapeutic group (ATC level 2)**	**Substance**	**Total (serious)**
Anaesthetics (N01)	Articaine	1(1)
	Bupivacaine	4(4)
	Fentanyl	24(24)
	Propofol	2(2)
	Sevoflurane	2(2)
	Sufentanil	4(4)
Total N01		37(37)
Analgesics (N02)	Buprenorphine	24(24)
	Codeine	14(14)
	Diamorphine	3(3)
	Dihydroergotamine	27(27)
	Ergotamine	2(2)
	Flupirtine	6(6)
	Frovatriptan	2(2)
	Hydromorphone	22(22)
	Metamizole	20(20)
	Methylergometrine	2(2)
	Methysergide	25(25)
	Morphine	6(6)
	Oxycodone	56(56)
	Paracetamol	42(42)
	Phenazone	8(8)
	Pizotifen	2(2)
	Propyphenazone	7(7)
	Sumatriptan	3(3)
	Tilidine	11(10)
	Tramadol	62(57)
Total N02		344(338)
Antiepileptic drugs (N03)	Carbamazepine	142(142)
	Clonazepam	13(13)
	Gabapentin	53(53)
	Lamotrigine	78(78)
	Levetiracetam	2(2)
	Oxcarbazepine	36(36)
	Phenytoin	6(6)
	Phenobarbital	4(4)
	Pregabalin	1510(1510)
	Topiramate	6(6)
	Valproate	17(17)
	Zonisamide	3(3)
Total N03		1870(1870)
Antiparkinson drugs (N04)	Amantadine	9(9)
	Apomorphine	45(45)
	Benserazide	1(1)
	Bromocriptine	26(26)
	Cabergoline	1(1)
	Carbidopa	43(43)
	Entacapone	39(39)
	Levodopa	44(44)
	Piribedil	1(1)
	Pramipexole	17(17)
	Procyclidine	7(7)
	Rasagiline	15(15)
	Rotigotine	1(1)
	Ropinirole	15(15)
Total N04		264(264)
Antipsychotics (N05A)	Amisulpride	1(1)
	Aripiprazole	19(19)
	Bromperidol	8(8)
	Chlorpromazine	7(7)
	Chlorprothixene	23(23)
	Clozapine	71(71)
	Flupentixol	1(1)
	Fluspirilene	2(2)
	Haloperidol	12(12)
	Levomepromazine	9(9)
	Lithium	18(18)
	Melperone	7(7)
	Olanzapine	23(23)
	Perphenazine	12(12)
	Pipamperone	3(3)
	Quetiapine	46(46)
	Risperidone	37(37)
	Sulpiride	7(7)
	Thioridazine	6(6)
	Tiapride	6(6)
	Zuclopenthixol	21(21)
Total N05A		339(339)
Anxiolytics (N05B)	Alprazolam	17(17)
	Bromazepam	14(14)
	Chlordiazepoxide	2(2)
	Clorazepate	9(9)
	Diazepam	19(19)
	Lorazepam	74(74)
	Oxazepam	8(8)
Total N05B		143(143)
Hypnotics and sedatives (N05C)	Butalbital	5(5)
	Clomethiazole	5(5)
	Flunitrazepam	1(1)
	Melatonin	4(4)
	Zaleplon	3(3)
	Zolpidem	14(14)
	Zopiclone	12(12)
Total N05C		44(44)
Antidepressants (N06A)	Agomelatine	29(29)
	Amitriptyline	11(11)
	Bupropion	7(7)
	Citalopram	38(38)
	Clomipramine	5(5)
	Duloxetine	27(27)
	Doxepin	1(1)
	Escitalopram	25(25)
	Fluoxetine	14(14)
	Imipramine	1(1)
	Mirtazapine	18(18)
	Nortriptyline	6(6)
	Opipramol	28(28)
	Paroxetine	21(21)
	Sertraline	22(22)
	Trimipramine	7(7)
	Venlafaxine	217(177)
Total N06A		477(437)
Psychostimulants (N06B)	Caffeine	16(16)
	Methylphenidate	61(61)
Total N06B		77(77)
Anti-dementia drugs (N06D)	Memantine	2(2)
	Rivastigmine	50(50)
Total N06D		52(52)
Parasympathomimetics (N07)	Disulfiram	11(11)
	Methylnaltrexone	2(2)
	Nicotine	91(91)
	Varenicline	1017(135)
Total N07		1121(239)
Total ATC group N		4766(2433)

**Table 4:** Serious adverse drug reactions reported for pregabalin by European consumers, 2007 to 2011

**Adverse drug reaction(s)**	**N**
Drug ineffective/drug effect decreased	83
Dizziness	78
Pain	61
Somnolence	56
Fatigue	54
Weight changes	47
Abdominal pain	39
Nausea	36
Headache	35
Vision blurred	28
Insomnia	24
Muscle spasms	24
Oedema	24
Gait disturbance	23
Myalgia	21
Hyperhidrosis	20
Appetite changes	18
Dry mouth	18
Malaise	17
Pruritus	17
Constipation	16
Disturbance in attention	16
Depression	15
Rash	15
Balance disorder	14
Memory impairment	14
Paraesthesia	14
Vertigo	14
Withdrawal syndrome	14
Accidental exposure	13
Diarrhoea	13
Feeling abnormal	13
Speech disorder	13
Anxiety	12
Arthralgia	12
Feeling drunk	12
Tremor	12
Eye swelling	11
Nasal congestion	11
Burning sensation	10
Erectile dysfunction	10
Urinary tract disorder	10
Vomiting	10
Others (n < 10)	492
Total	1510

**Table 5:** Serious adverse drug reactions reported for varenicline by European consumers, 2007 to 2011

**Adverse drug reaction(s)**	**N**
Musculoskeletal pain	9
Sleep disorder	7
Chest discomfort/pain	7
Depression	6
Suicidal behaviour/ideation	6
Nausea	5
Rash	4
Aggression	3
Mood altered/mood swings	3
Feeling abnormal	3
Headache	3
Oropharyngeal blistering/pain	3
Anxiety	2
Hallucination	2
Tearfulness	2
Fatigue	2
Pyrexia	2
Epilepsy	2
Movement disorder	2
Muscle spasms/weakness	2
Abdominal discomfort/pain	2
Erythema	2
Hypersensitivity	2
Others (n < 2)	53
Total	135

## Discussion

This is the first study to systematically analyse ADRs for nervous system medications reported by consumers to the EV database. Almost all ADRs, except for those reported for parasympathomimetics, were serious and several fatal cases were reported. Reported ADRs were predominantly of the type nervous and psychiatric disorders and general disorders. The majority of ADRs were reported for pregabalin, varenicline and venlafaxine.

### ADRs by type and seriousness

The most frequently reported ADRs for nervous system medications were of the type nervous and psychiatric disorders and this finding was expected due to the mechanism of action of the reported nervous system medications. Additionally, a large number of ADRs of the type general disorders and administration site conditions and gastrointestinal disorders were reported, and this finding was also in line with results in previous consumer studies [5-13]. More than one half of reported ADRs were serious, however this reporting pattern was not surprising, since countries were not requested to report non-serious ADRs to the EV database during the study period [16].

### ADRs by therapeutic groups

The largest number of ADRs was reported for antiepileptics and antidepressants, which can be explained by the frequent use of these medications in adults [20]. A high number of ADRs were reported for varenicline but only few were serious. In 2007, based on consumer reports in the USA, there was a high media attention on the increased risk of serious ADRs such as suicidal ideation and occasional suicidal behaviour, erratic behaviour and drowsiness reported for varenicline leading to black box warnings in the USA (July 2009) [21]. The ADR signal was later confirmed in a meta-analysis [22]. The high number of ADRs reported for varenicline by European consumers could have been stimulated by this media attention; however, the majority of reported ADRs were non-serious. For pregabalin a large number of the ADRs “drug ineffective/drug effect decreased” were reported, probably because this side effect can easily be assessed, and is very obvious compared to many other types of ADRs. To evaluate whether ADRs reported for pregabalin and varenicline can act as early warning for new ADR signals more in-depth analysis of the ADR reports should be conducted.

### Strengths and limitations of this study

The strength of this study is that data comprised all ADRs reported by consumers in Europe, which were forwarded to the EV database during a five-year period and present in the database by March 2012. A major limitation to this study is that we do not know to which extent the causality of these ADRs can be confirmed, and this has implications for the interpretation of the findings [2]. The value of consumer reports in detection of new ADR signals remains unclarified due to the lack of information about causality. In this study, we did not evaluate the validity of the consumer reports since we only had access to the data entered into the EV database and not the original reports. Spontaneous reporting systems suffer from various barriers, such as incomplete recognition of ADRs, administrative barriers to reporting and low data quality, all of which may result in under-reporting of important serious and rare events [2]. ADRs that are non-serious or already known may be over-reported; however, this study provides information on reported ADRs, and this information contributes to broadening the knowledge on medicine safety. Before July 2012 countries were only obliged to report serious consumer reports to EV, which may explain the large number of serious ADRs found, and the low number of non-serious consumer reports. Therefore there may be additional non-serious consumer ADR reports present in the regulatory agencies. With the new pharmacovigilance regulation that came into force in July 2012 the share of serious consumer reports in EV will probably decline although the total number of consumer reports is expected to increase.

Hence, it is not possible to generalize from data reported to the EV database to the other EU member states. Spontaneous reports are an important source of information about new and previously unrecognized ADRs, and the value of spontaneous reporting schemes lies in their ability to act as hypothesis-generating procedures [2]. Therefore, EMA should continue to systematically survey and analyse ADRs reported by consumers in order to signal previously unknown ADRs. Another important issue to be investigated in future studies is to which extent individuals suffering from ADRs later recover from the reported reactions.

## Conclusion

The majority of ADRs from nervous system mediations reported by consumers that were identified from the EudraVigilance database were serious. The value of consumer reports in pharmacovigilance still remains unclarified.

## Competing interests

The authors have not received reimbursements, fees, funding, or salary from an organization that may in any way gain or lose financially from the publication of this manuscript, either now or in the future. The authors do not hold stocks or shares in an organization that may in any way gain or lose financially from the publication of this manuscript. The authors do not hold or plan to apply for any patents relating to the content of the manuscript. The authors have not received reimbursements, fees, funding, or salary from an organization that holds or has applied for patents relating to the content of the manuscript. The authors declare no other non-financial competing interests.

## Authors’ contribution

LA and EHH designed the study, analysed data and wrote the first version of the manuscript. LA carried out the sampling. Both authors saw and approved the final version of the manuscript. No sources of funding were used to assist in the preparation of this study.

## Pre-publication history

The pre-publication history for this paper can be accessed here:

http://www.biomedcentral.com/2050-6511/14/30/prepub
